# Reconstructing complex network for characterizing the time-varying causality evolution behavior of multivariate time series

**DOI:** 10.1038/s41598-017-10759-3

**Published:** 2017-09-05

**Authors:** Meihui Jiang, Xiangyun Gao, Haizhong An, Huajiao Li, Bowen Sun

**Affiliations:** 1 School of Humanities and Economic Management, China University of Geosciences, Beijing, 100083 China; 2 Key Laboratory of Carrying Capacity Assessment for Resource and Environment, Ministry of Land and Resources, Beijing, 100083 China; 3 Key Laboratory of Strategic Studies, Ministry of Land and Resources, Beijing, 100812 China

## Abstract

In order to explore the characteristics of the evolution behavior of the time-varying relationships between multivariate time series, this paper proposes an algorithm to transfer this evolution process to a complex network. We take the causality patterns as nodes and the succeeding sequence relations between patterns as edges. We used four time series as sample data. The results of the analysis reveal some statistical evidences that the causalities between time series is in a dynamic process. It implicates that stationary long-term causalities are not suitable for some special situations. Some short-term causalities that our model recognized can be referenced to the dynamic adjustment of the decisions. The results also show that weighted degree of the nodes obeys power law distribution. This implies that a few types of causality patterns play a major role in the process of the transition and that international crude oil market is statistically significantly not random. The clustering effect appears in the transition process and different clusters have different transition characteristics which provide probability information for predicting the evolution of the causality. The approach presents a potential to analyze multivariate time series and provides important information for investors and decision makers.

## Introduction

Reconstructing complex network from time series has been widely applied to characterize nonlinear dynamic behavior in a time-dependent system from the observed time series. Typically, scholars detect the dynamic features from single time series or the relationships between two different time series^1–4^. However, in a real complicated system, there are usually more than one or two time series which intertwine and interact with each other. The evolution of the interaction behavior between multivariate time series can help us to uncover the hidden dynamic interaction information. For coping with the increased complexity, new complex network methods are required to transfer the evolution of the time-varying relationships of multivariate time series to a complex network and to explore the underlying evolution mechanism affected by the multi-information fusion^5^.

Previous studies, in the last decade, have witnessed the success and effectiveness of complex network in analyzing nonlinear characteristics of time series in many fields, such as engineering, management, economic and biology^6–13^. Zhang and Small first proposed that univariate time series can be transformed to networks^14^. Lacasa *et al*. proposed the visibility graph algorithm to abstract the time point in time series to simple patterns and relationship^15^. It provided a new perspective for research on the complexity of time series by turning time series into graphs. Moreover, Gao *et al*. developed a novel multiscale limited penetrable horizontal visibility graph (MLPHVG) to analyze nonlinear time series^16^. Meanwhile, the concept of coarse graining in the phase space is widely used to describe the fluctuation of time series. After the coarse graining process, the volatility patterns of time series are expressed as modes. An *et al*. studied the complexity of the autocorrelation modes of univariate time series^17^. Based on the concept of the modes, Gao *et al*. built models and analyzed the volatility modes of the comovement and the cross-correlation between bivariate time series^1, 3^. Recently, some studies gradually focus on analyzing multivariate time series by taking the correlations or distances between time series as the relationship sets^18–21^.

Based on existing contributions above, there is still a big challenge to uncover the richer dynamic information in the fluctuation of inner relationships of multivariate time series. The correlation and distance between time series only reflect limited information. It is necessary to find a more appropriate and rigorous method to define the relationship between time series. In certain fields, like economics and neuroscience, Granger causality test has been widely used to measure the relationships between time series^22–25^. One of the advantages of Granger causality test is that it can statistically measure the extent to which one time series explains the change of another time series in the future^26–28^, and detect the directed links between time series. The most existing works focus on the long-term causality between time series, ignoring the dynamic adjustment mechanism of short-term fluctuation toward to the long-term equilibrium^28–31^. Recently, some studies developed various time-varying causality methods to investigate the dynamical linkages between some economic variables, such as stock market and exchange rate, spot and futures crude oil prices, and crude oil and stock markets^32–36^. However, these researches still essentially analyzed the causalities between bivariate time series, lacking the systematic perspective to uncover the hidden dynamic interaction information between multivariate time series, and to understand the evolution mechanism of the complicated system.

In this paper, we define the two-dimensional matrix which contains all causalities between any two time series as the causality patterns. If a time series is divided into several fragments (sub-periods), the causality pattern of time series in the long term can be described by the union by all the short-term causality patterns in sub-periods. The evolution of the causality patterns forms a time-varying process of transition, which can help us learn the fluctuation of the relationships between time series. In the process of transition, there are different types of causality patterns that convert to each other and form a multivariate time-varying causality transition network, which will reveal the structure characteristics of the evolutionary behavior of the relationships between multivariate time series.

The main purpose of this paper is to propose a novel network model to detect the nonlinear dynamic features from the evolution behavior of the time-varying causalities between multivariate time series. First, we test the short-term causalities between any two time series and map the transition behavior of the causality patterns into the network. Then, we study the transition characteristics of the causality patterns using the complex network analytical approach, including the distribution of the causality patterns, the transition patterns and the clustering effect in the transition process.

## Network Model

### The Granger causality test

The Granger causality test was proposed by Clive W. J. Granger^37^. For given stationary time series *x*(*t*), the autoregressive model is given by:

1 $$x(t)=\sum _{i=1}^{m}{\alpha }_{i}x(t-i)+{\varepsilon }_{x}(t)$$

*ε*_*x*_(*t*) denotes the residual error and *m* denotes the lag intervals for *x*(*t*). Now we suppose we have another stationary time series *y*(*t*). If *x*(*t*) is influenced by both the past value from *y*(*t*) and itself, the model can be given by:

2 $$x(t)=\sum _{i=1}^{p}{\alpha }_{i}x(t-i)+\sum _{j=1}^{q}{\beta }_{i}y(t-j)+{\varepsilon }_{x|y}(t)$$

It forms a vector autoregression model around *x*(*t*). *p* is the lag length of *x*(*t*) and *q* is the lag length of *y*(*t*). In this paper, we use the Bayesian Information Criterion (BIC) to automatically select *p* and *q*. If *x*(*t*) is influenced by both the past value from *y*(*t*) and itself, we can set a null hypothesis: *H*_0_ = *β*_1_ = *β*_2_ = …… = *β*_*m*_ = 0, which means *y*(*t*) does not Granger cause *x*(*t*). To test this hypothesis, we can use the homogeneity test of variances as follows:

3 $$F=\frac{(RS{S}_{r}-RS{S}_{u})/q}{RS{S}_{u}/(T-p-q-1)} \sim F(q,T-p-q-1)$$

*RSS*_*t*_ is the residual sum of squares without the lagged variable *y*(*t*). *RSS*_*u*_ is the residual sum of squares with the lagged variable *y*(*t*). *T* denotes the sample size.

If $F\ge {F}_{\alpha }(q,\,T-p-q-1)$, we reject the null hypothesis, which means *y*(*t*) Granger causes *x*(*t*). In this paper, we choose the significance level *α* = 0.05 as the causality threshold to determine the causality among time series. It means if *p* value, which can be calculated according to *F-*statistic, ≤ 0.05, *y*(*t*) Granger causes *x*(*t*).

### The construction of the multivariate time-varying causality transition network

To obtain the time-varying Granger causality between time series, we use the sliding window to divide time series into several sub-periods. Compared with dividing time series into different individual time periods, the advantage of sliding window is that they contain the features of memory and transitivity^1, 17, 38^. In this paper, the length of a sliding window should satisfy three conditions: (1) Data in each sub-period should be stationary, which is one of the precondition of Granger causality test. (2) The length of sliding windows should serve the needs of the analysis^1, 3, 4^. If the goal is to study the transition characteristics of the causality patterns in the short term, the length can be set to a smaller value. If the goal is to understand the transition characteristics in the long term, the length can be set to a larger value. (3) The diversity of the causality patterns and transitions among them should be guaranteed. As we know, with the increase in the length of sliding windows, data in the sub-period will be more similar to the original time series.

Let *w* represents the length of a sliding window. First, we choose day *t* as start point and get *sub-period*_*t*_, which is from day *t* to day *t* + (*w* − 1). Then, we choose day *t* + 1 as start point and get *sub-period*_t+1_ which is from day *t* + *1* to day *t* + (*w - 1* + *1*). By that analogy, we can obtain a series of sub-periods. Next, we construct the multivariate time-varying causality transition network following three steps.

Step 1: Defining the Granger causality between any two time series. In a sub-period, we test the Granger causality between any two time series. If time series *j* Granger causes time series *i*, *GC*_*ij*_ = 1. If time series *j* does not Granger cause time series *i*, *GC*_*ij*_ = 0.

4 $$G{C}_{ij}=\{\begin{array}{l}1,\,j\,Grangers\,Cause\,i\,\\ 0,\,j\,doesn\mbox{'}t\,Granger\,Cause\,i\end{array}$$

Step 2: Defining the causality patterns. After determining the Granger causality between any two time series, the Granger causalities of all pairs of time series form a *n* × *n* matrix.

5 $$GC{M}_{t}=[\begin{array}{ccc}G{C}_{11}(t) & \cdots  & G{C}_{1n}(t)\\ \vdots  & \ddots  & \vdots \\ G{C}_{n1}(t) & \ldots  & G{C}_{nn}(t)\end{array}]$$

In this paper, we choose four time series, so *n* = 4. Although previous studies found that time series can be predicted by its own past (self-entropy)^39, 40^, however, our method is mainly to analyze the evolution process of the interactions between different time series, so we don’t include the causality between a time series and itself in this paper. Thus, the Granger Causality matrix can be shown in Table 1.

**Table 1:** The 4 × 4 Granger Causality Matrix.

	S_1_	S_2_	S_3_	S_4_
S_1_		*GC*_12_	*GC*_13_	*GC*_14_
S_2_	*GC*_21_		*GC*_23_	*GC*_24_
S_3_	*GC*_31_	*GC*_32_		*GC*_34_
S_4_	*GC*_41_	*GC*_42_	*GC*_43_	

**Figure 1:**
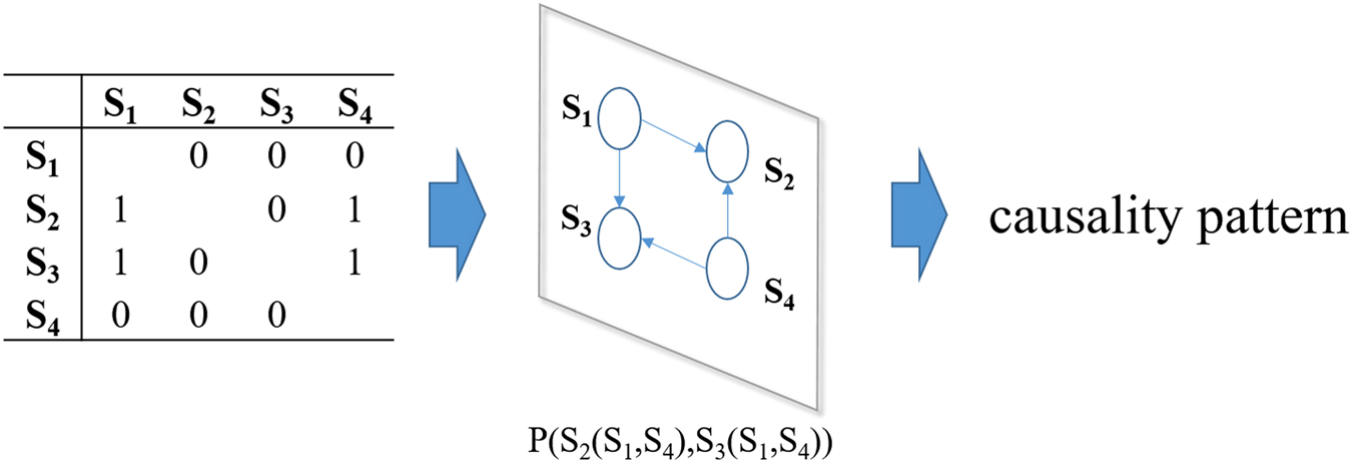
The definition of the causality patterns.

To better represent the Granger causality among time series as the causality patterns, we can turn the matrix into an expression. For example, if *GC*_*ij*_ = 1, it means time series *j* Granger causes time series *i* and can be signified as *i*(*j*). Then if the existing Granger causalities in a sub-period are S_2_(S_1_,S_4_) and S_3_(S_1_,S_4_), the causality pattern would be represented as P(S_2_(S_1_,S_4_), S_3_(S_1_,S_4_)) (Fig. 1).

**Figure 2:**
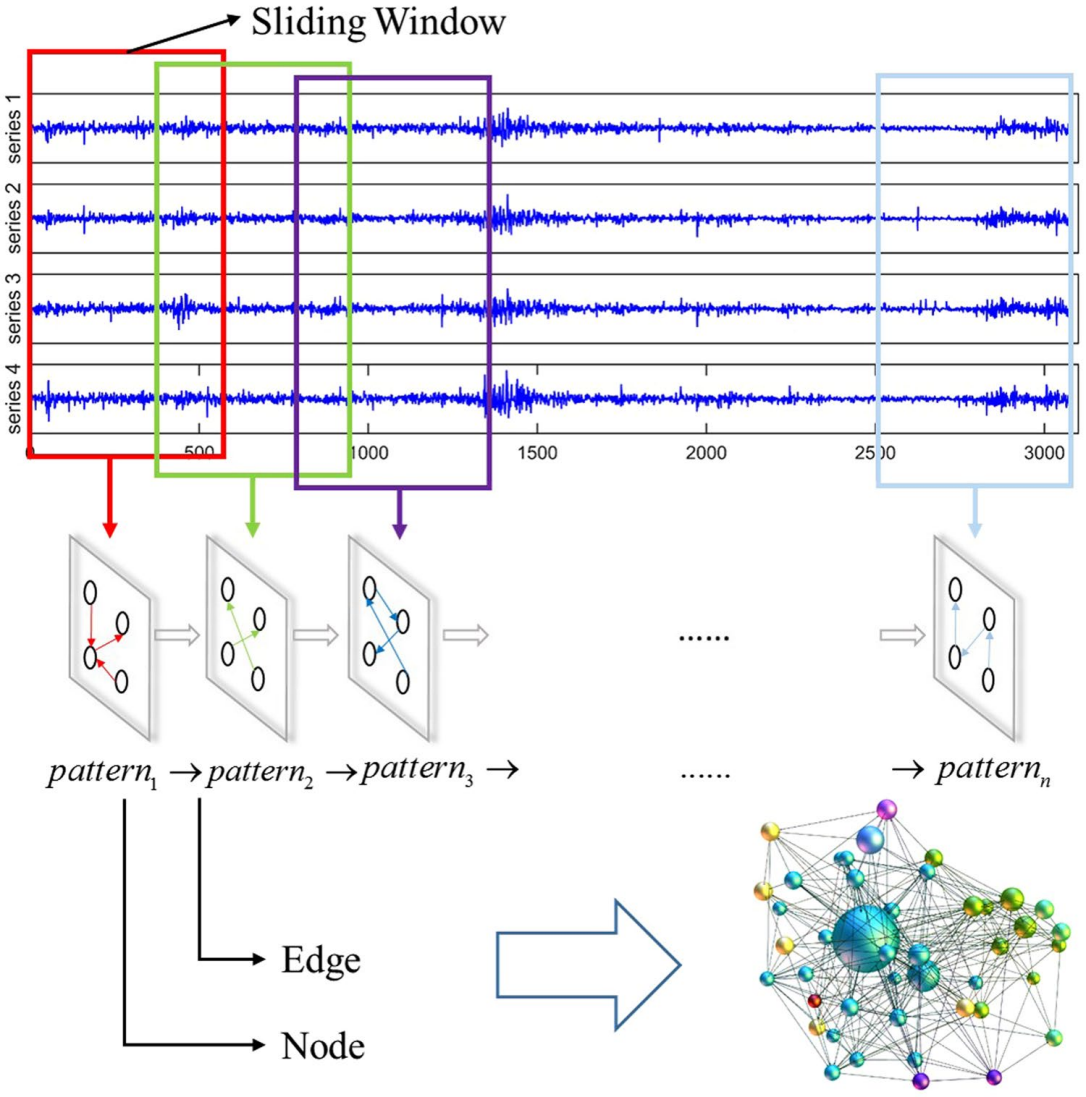
Schematic illustration of constructing the multivariate time-varying causality transition network.

Step 3: Constructing the multivariate time-varying causality transition network. As the sliding windows move, we obtain a series of causality patterns. One causality pattern is converted to another as time goes by: *pattern*_1_ → *pattern*_2_ → *pattern*_3_ → …… → *pattern*_*n*_ (*n* = *T − w* + *1*). Because the conversion between two types of causality patterns would repeat in the transition process, the trajectory of the conversion among causality patterns forms a network. To map the transition network, we take the causality patterns as nodes and the succeeding sequence relations between the causality patterns as edges. The weight of an edge is the frequency of the transition between two types of causality patterns. The process of building the multivariate time-varying causality transition network is shown in Fig. 2.

### Sample data

In this paper, we focus on the relationships between multivariate time series. As the precondition of our work, there should be the theoretical and realistic relationships between time series. Based on this, we choose four real time series to study the transition behavior of the causality patterns from the international oil market. They are the West Texas Intermediate spot price (W), the Brent spot price (B), the Dubai spot price (D) and the Minas spot price (M). These four international crude oil benchmark prices are authoritative in the international oil market and represent four oil market in different regions^41^. The cointegration or causal relationships between them have been studied from different perspectives by other scholars^32, 42^. We use the daily price because the shock transmission among oil prices is very fast. The data cover the period from January 2, 2003 to December 31, 2015.

*P*_*t*_ denotes the daily closing price of crude oil on day *t*. The daily price return of crude oil is calculated as follows:

6 $${r}_{t}=\,\mathrm{ln}({P}_{t})-\,\mathrm{ln}({P}_{t-1})$$

The results of the stationarity tests of the returns of four oil prices are shown in Table 2.

**Table 2:** Results of the stationarity tests.

	ADF		PP	
	t-Statistic	Probability	t-Statistic	Probability
*r*_*Brent*_	−53.38	0.0001	−53.39	0.0001
*r*_*Dubai*_	−61.02	0.0001	−60.92	0.0001
*r*_*Minas*_	−56.96	0.0001	−56.95	0.0001
*r*_*WTI*_	−57.96	0.0001	−57.92	0.0001

Note: Daily data for the period from 2 January 2003 to 31 December 2015. The t-Statistic is the statistic for the test of the stationarity. Probability refers to the p-value of the t-Statistic. p-value < 0.01 indicates the rejection of the null hypothesis for the associated statistical tests at the 1% level. We choose ADF(Augmented Dickey–Fuller test) and PP(Phillips–Perron test) as the stationarity test methods.

## Results

### Statistical features of the transition process

**Figure 3:**
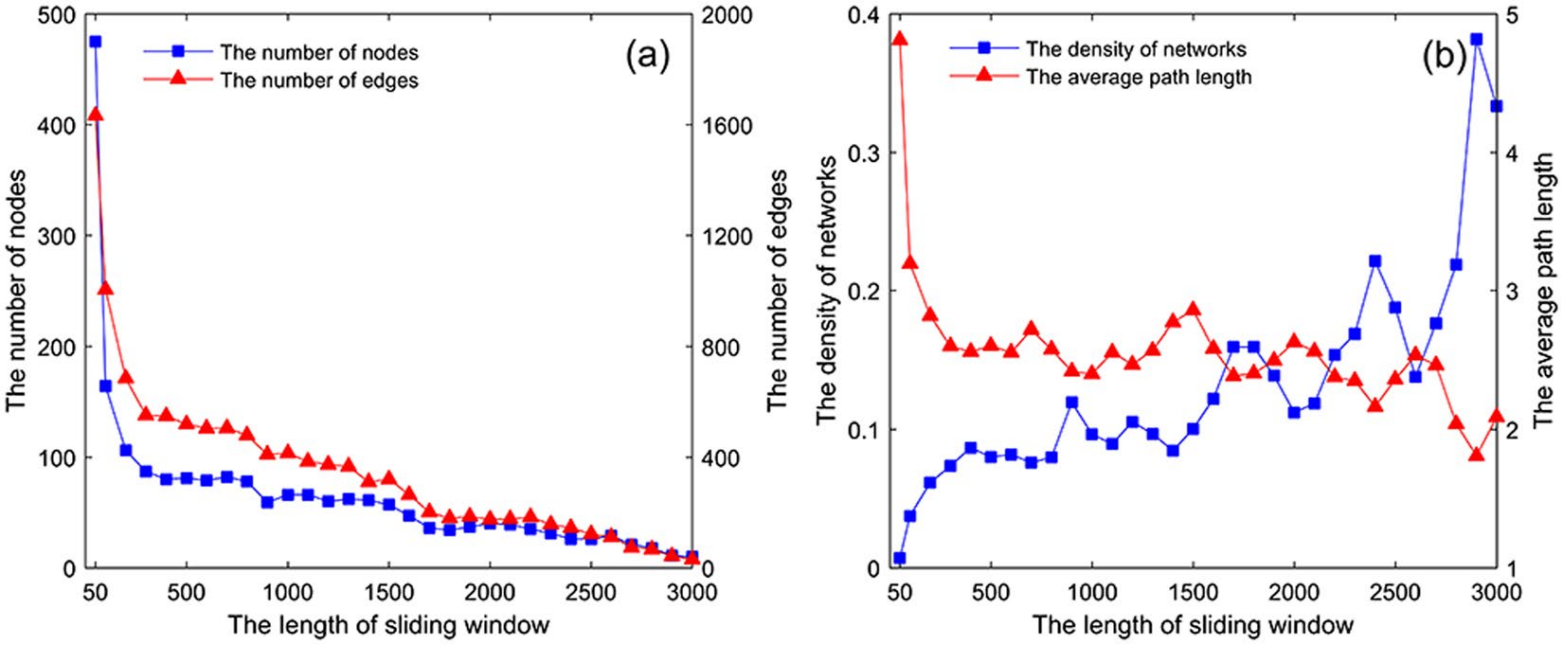
Sensitivity analysis. (**a**) Number of nodes and edges for different lengths of sliding window. (**b**) The density of networks and the average path length for different lengths of sliding window.

Before constructing the multivariate time-varying causality transition network, we do the sensitivity analysis to select the appropriate length of sliding window. With the increasing of the length of the sliding window, it can be seen in Fig. 3(a) that the number of nodes and edges both decrease, while the densities of networks increase and the average path length of networks decrease (Fig. 3(b)). Combining the results of the sensitivity analysis with the three conditions we have discussed above, in this paper, we set the size of a sliding window for 50 days. We also choose other 3 kinds of length of sliding window, i.e., 100 days, 500 days and 1000 days, to analyze the dynamic features of the multivariate time-varying causality transition process, please see the detail in the Supplementary Information.

In the full period, the Granger causalities existing in four time series (Brent spot price, Dubai spot price, Minas spot price, WTI spot price) are B(W), D(B,M,W) and M(B,W), which means that W Ganger causes B; B, M and W Granger cause D; and B and W Granger cause M (Table 3). The results make us understand the Granger causality among the international benchmark oil prices in the long term. Time series change over time, however, so the relationships between time series also change over time.

**Table 3:** The Granger Causality Matrix in the full period.

	B	D	M	W
B		0	0	1
D	1		1	1
M	1	0		1
W	0	0	0	

After constructing the multivariate time-varying causality transition network, we obtain 230 nodes and 1021 edges. Each node represents a causality pattern. Therefore, there are 230 types of causality patterns in the selected period of data. With the help of the sliding windows, we divide the time series into 3032 fragments, meaning that there should be 3032 causality patterns. However, there are only 230 types of causality patterns in the network. The number of the edges (1021) in actuality is also much fewer than the number (3031) in theory. Thus, the evolution of the time-varying causalities between different time series has some characteristics of complexity including concentricity, repetitiveness and regression.

### The key causality patterns in the transition process

To recognize the key causality patterns in the transition process, we use the weighted degree to measure the importance of causality patterns. The weighted degree is a comprehensive index to measure the importance of the nodes which considers not only the number of its adjacent nodes but also the weight connecting to its adjacent nodes. Weighted degree is calculated as follows:

7 $${w}_{i}={w}_{i}^{in}+{w}_{i}^{out}=\sum _{j\in {N}_{i}}{w}_{ji}+\sum _{j\in {N}_{i}}{w}_{ij}$$

*N*_*i*_ denotes the set of the nodes connecting to node *i*. *w*_*ij*_ denotes the weight of the edge from node *j* to node *i*. *w*_*ij*_ denotes the weight of the edge from node *i* to node *j*. As we defined, we take the frequency of the transition between two types of causality patterns as the weight of an edge.

**Figure 4:**
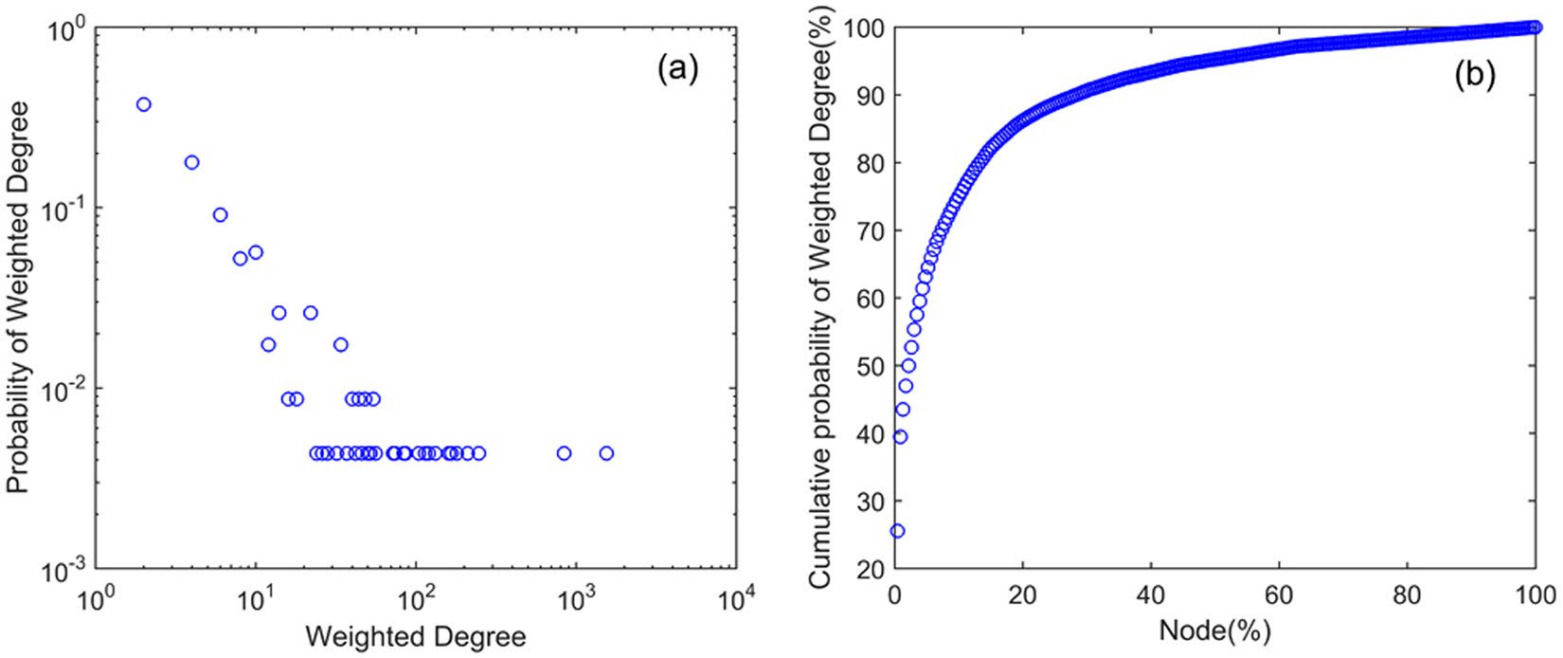
The distribution of the weighted degree.

Weighted degree of the nodes obeys power law distribution $p(w) \sim {w}^{-\lambda }$ (Fig. 4(a)). This means there are a few key causality patterns in the network. The cumulative distribution of the nodes’ weighted degree shows that 20% of the nodes shoulder 85% of the weight (Fig. 4(b)). This implies that a few types of causality patterns play a major role in the process of the transition and that international crude oil market is statistically significantly not random. The oscillations of the market are driven by a few types of causality patterns. After ranking the nodes by weighted degree, we can recognize the key causality patterns (Table 4). Comparing to the long-term causality pattern P(B(W), D(B,M,W), M(B,W)), some long-term causalities don’t appear statistically frequently in some periods, like D(M). Meanwhile, there are some short-term causalities which aren’t the significant causalities in the long term, like B(W). Due to the competition among different oil producing regions, the production and supply in different regions would have influence on each other. The massive and varying market information affect the market all the time. Thus, the state of the market would not keep exactly same with that in the long term. Recognizing the special short term causalities can provide the useful information to keep the decisions more dynamical and well-adapted.

**Table 4:** Top 10 causality patterns and their weighted degree in the dynamic Granger causality network.

Node	Weighted Degree	Percentage accounts for total Weighted Degree
P(B(W),D(B,W),M(B,W))	1550	0.2557
P(D(B,W),M(B,W))	842	0.1389
P(B(W),D(B,W),M(B,W),W(B))	247	0.0407
P(B(W),D(B,W),M(B,D,W))	210	0.0346
P(B(D,M,W),D(B,W),M(B,W))	180	0.0297
P(D(B,W),M(B,D,W))	166	0.0274
P(B(W),D(B,M,W),M(B,D,W))	160	0.0264
P(B(W),D(B,M,W),M(B,W))	132	0.0218
P(B(W),D(B,W),M(B,W),W(B,D,M))	120	0.0198
P(D(B,W),M(W))	114	0.0188

### The transition patterns in the transition process

**Figure 5:**
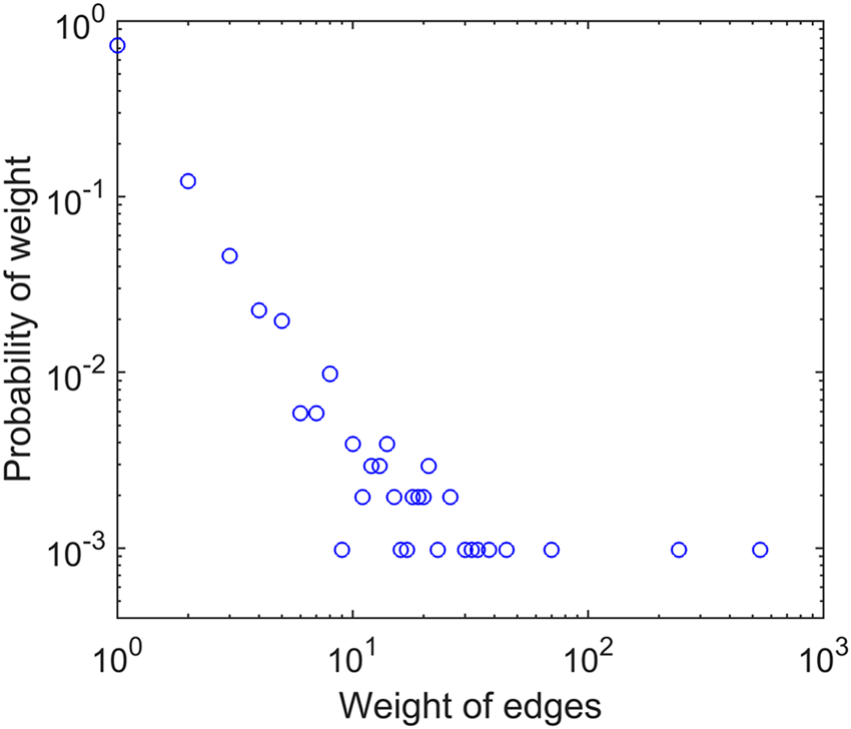
The distribution of weight of edges.

After recognizing the key causality patterns in the transition process, we find that a complete transition unit consists of not only nodes but also edges between nodes. A basic transition unit, which can be called the transition pattern, should contain two nodes and their edge: ${T}_{ij}=\{patter{n}_{i},patter{n}_{j}\}$. *T*_*ij*_ represents the transition process by which *pattern*_*i*_ converts to *pattern*_*j*_. Different transition patterns superimpose together and form the complete transition process. In this paper, we divided time series into 3032 fragments, so there are 3021 transition patterns theoretically. However, there are only 1021 weighted and directed edges in actuality. According to Fig. 5, the distribution of the weight of the transition patterns follows power law. Thus, a few certain key transition patterns exit in the transition process. These phenomena show that the transition among causality patterns is not a random process while it follows the rules in which the transition process is controlled by some key causality patterns. More specifically, these key causality patterns tend to convert to themselves. For example, P(B(W),D(B,W),M(B,W)) → P(B(W),D(B,W),M(B,W)).

**Figure 6:**
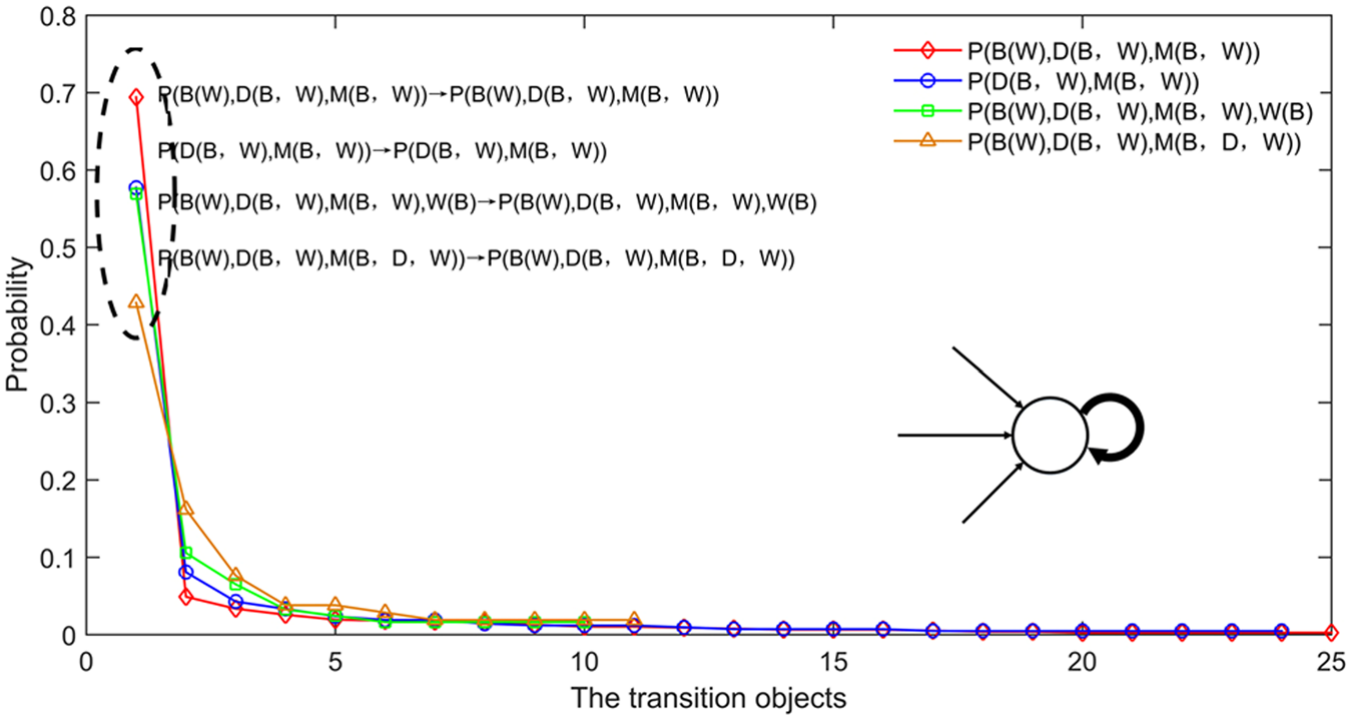
The transition probabilities of the key causality patterns. We choose the edges with weight > 1.

As shown in Fig. 6, the key causality patterns have many transition objects. It means that the key causality patterns convert to several causality patterns including themselves. However, only a few transition objects have high transition probability. The transition object of a key causality pattern which has the highest transition probability is the key causality pattern itself. Other causality patterns tend to convert to these key causality patterns and these key causality patterns tend to convert to themselves. Thus, a few key causality patterns have important regulating effect in the transition process and induce the transition process to show the characteristics of the regression. In other words, the key causality patterns can control the randomness of the transition process.

### The clustering effect in the transition process

**Figure 7:**
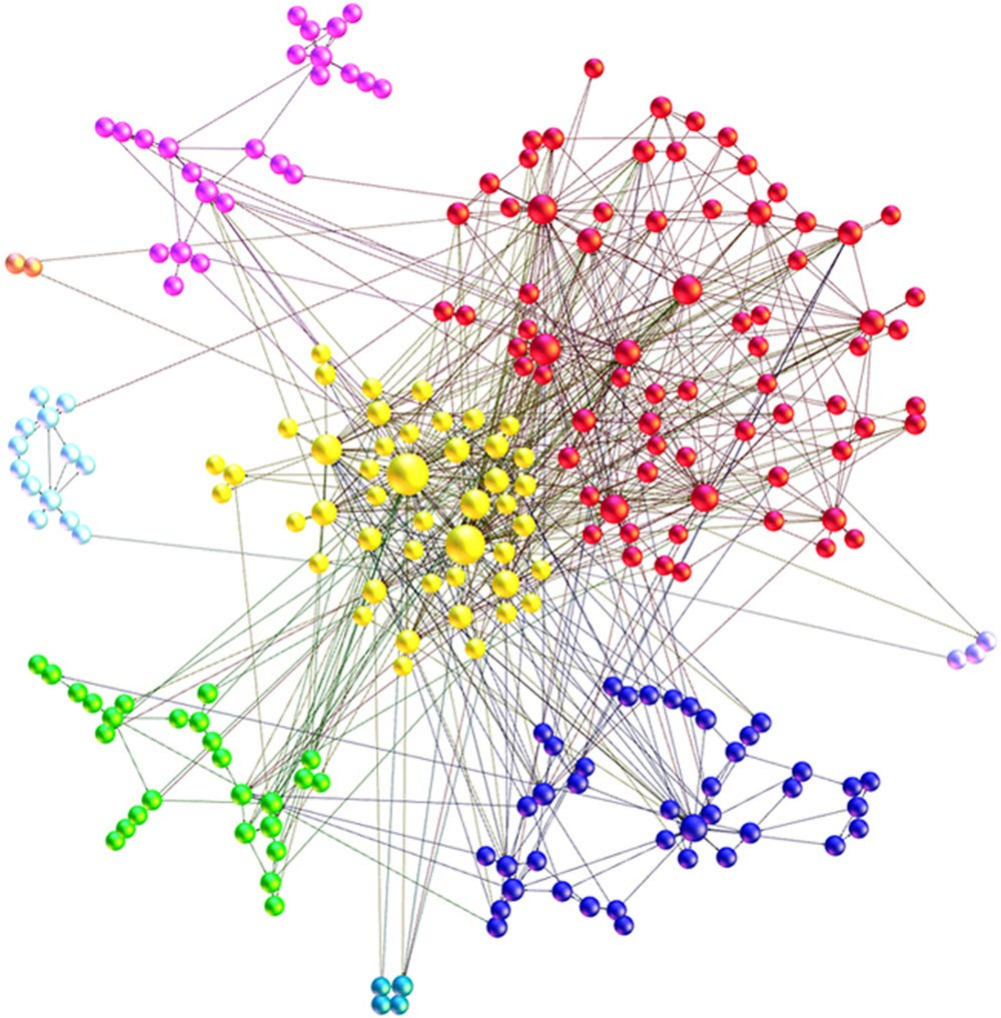
The clustering effect in the multivariate time-varying causality transition network. Note: red-cluster 1(31.3%), yellow-cluster 2(20%), blue-cluster 3(17.39%).

Based on the study of the key causality patterns, we found that some causality patterns convert to each other more frequently rather than converting to other causality patterns. This phenomenon makes some causality patterns and their relations form some clusters. The causality patterns in a cluster connect relatively closely to each other, so each cluster represents a special transition type. The analysis of clusters can provide some reference information for predicting the development of the relationship among time series. Blondel provided an algorithm to divide the network into clusters accurately and efficiently^43^. The algorithm is based on modularity in the networks^44^ (For details on the algorithm of dividing clusters, please see ref. 38). Applying the algorithm, we divide the network into 14 clusters. The amount of the nodes among the three clusters accounts for 68.69%. Therefore, the three clusters are the main clusters that can represent the entire network (Fig. 7).

**Figure 8:**
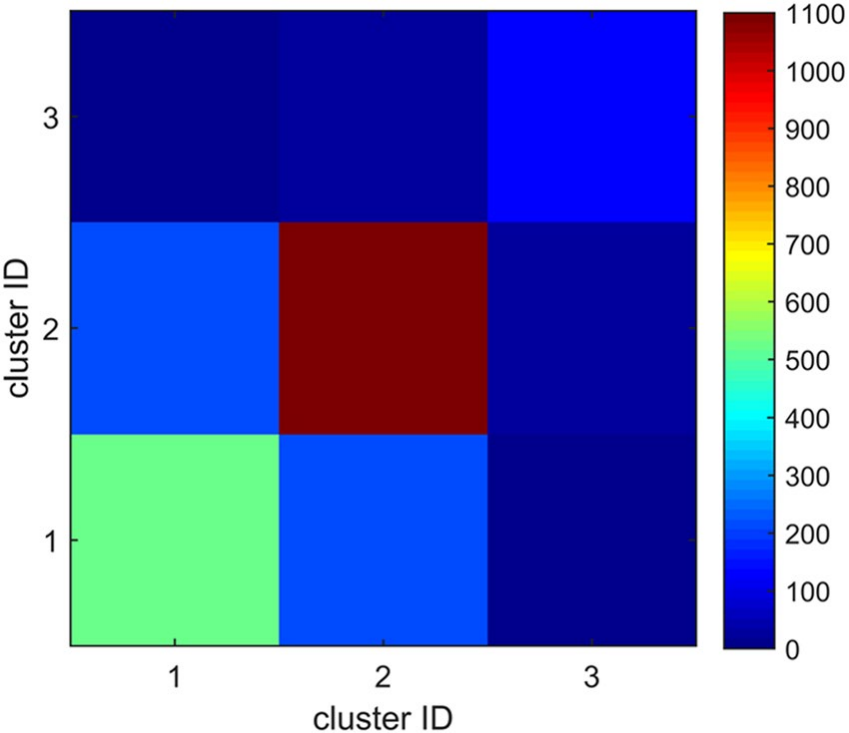
The distribution of transition abilities among the clusters.

One cluster has different transition ability to different clusters. The transition ability from cluster *m* to cluster *n* can be calculated as^1^: $T{A}_{mn}=\sum _{i\in m,\,j\in n}{w}_{ij}$, where *w*_*ij*_ is the weight from node *i* to node *j*. The distribution of the transition abilities between any two clusters is shown in Fig. 8. According to the distribution of the transition abilities, the transition abilities in one cluster are larger than the transition abilities between two different clusters, which proves that the cluster partition is effective. The clusters are independent and represent different transition types of the causality patterns.

**Figure 9:**
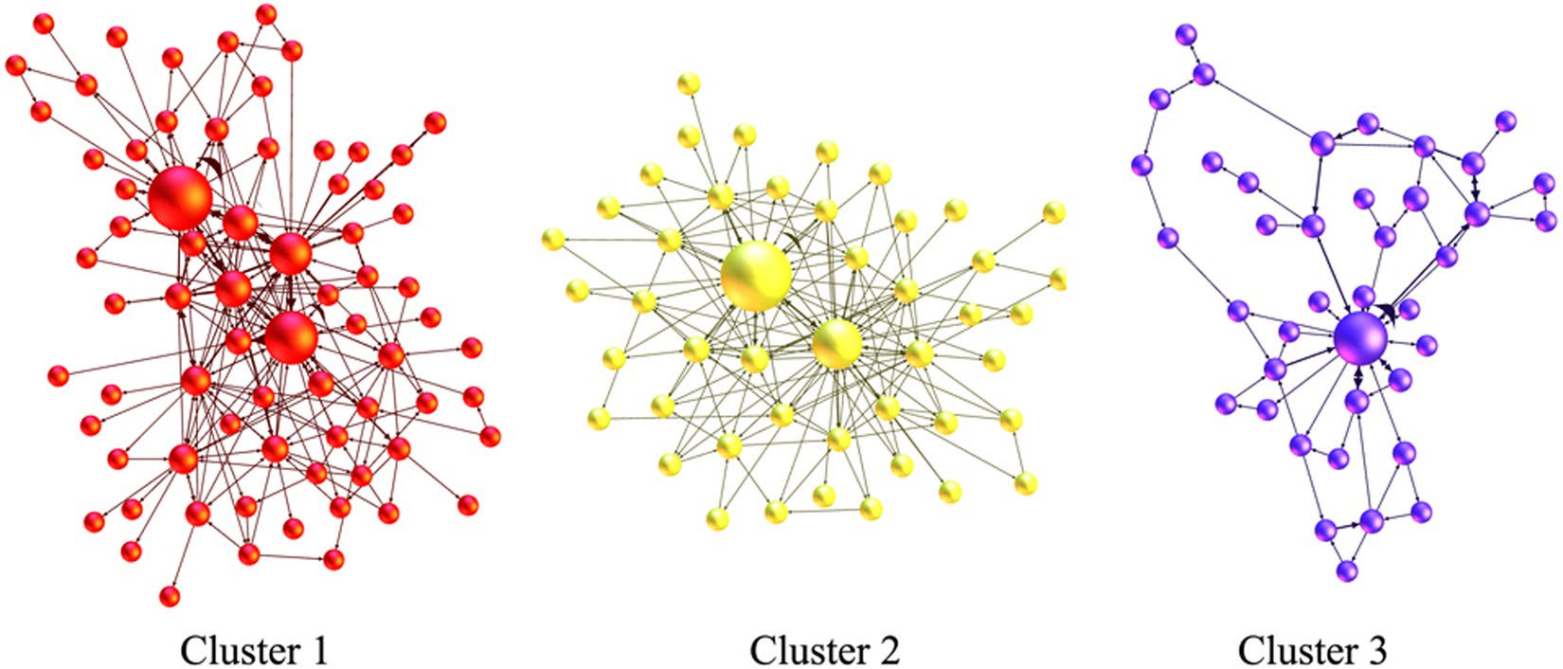
The three sub networks formed by three major clusters.

Extracting the three major clusters from the multivariate time-varying causality transition network, each cluster forms one sub network (Fig. 9). Analyzing the structure of three sub networks can help us understand the characteristics of three major clusters. According to Table 5, cluster 1 has the most nodes and edges. Cluster 2 has fewer nodes and edges than cluster 1. Cluster 3 has the least nodes and edges. It means there are many causality patterns and they frequently convert to each other in cluster 1. In cluster 3, there are both fewer causality patterns and conversion among the causality patterns. The average path length of cluster 2 is the shortest but the average clustering coefficient is the largest. It means the network structure of cluster 2 is the tightest. On the contrary, the average path length of cluster 3 is the longest while the average clustering coefficient is the smallest. It means the network structure of cluster 3 is loosest. Thus, the transition of the causality patterns is multidirectional and unpredictable in cluster 2 whereas the transition of the causality patterns in cluster 3 is the chain transition that has a certain direction.

**Table 5:** The structure characteristics of three major clusters.

	Cluster 1	Cluster 2	Cluster 3
The number of nodes	72	46	40
The number of edges	244	212	80
The average clustering coefficient	0.256	0.516	0.126
The average path length	2.957	2.408	4.104

**Figure 10:**
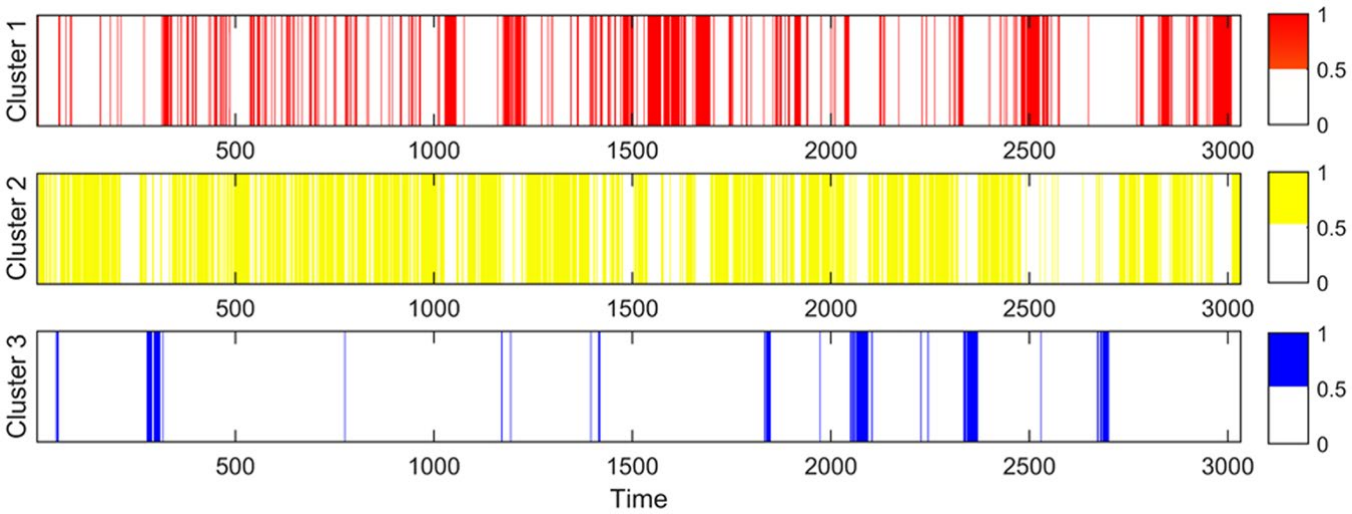
The distribution of the causality patterns in three major clusters over time.

The distribution of the transition process among the causality patterns in the three major clusters over time is shown in Fig. 10. According to Fig. 10, the causality patterns cluster at time 1600, time 2500 and time 3000 in cluster 1. The distribution of the causality patterns in cluster 2 is denser than that in cluster 1. Considering that nodes in cluster 2 are less than that in cluster 1, the causality patterns convert to others more frequently in cluster 2. The distribution of the causality patterns in cluster 3 is relatively sparse and clusters at time 300, time 2100, time 2400 and time 2700.

## Discussion and Conclusion

In this paper, our major aim is to explore the evolution characteristics of the time-varying relationships between multivariate time series by transferring the dynamic relationship transition behavior to complex network. We firstly tested the short-term causalities between any two time series and defined the causality pattern which can describe the causalities between multivariate time series. Then, we transformed the transitions between causality patterns to complex network. We analyzed the structure characteristics of the multivariate time-varying causality transition network to understand the dynamic behavior of the fluctuation of the relationships between multivariate time series, including the distribution of the causality patterns, the transition patterns and the clustering effect in the transition process.

In this paper, we chose four crude oil prices as the sample data. The results showed that the method is helpful to find the evolution features of the structure of the international crude oil market. First, the method can recognize a few key causality patterns in the transition process. These statistically frequent causality patterns reflected the main states of market. Some short-term relationships between different time series, which were caused by complicated information from outside environment, should be equally concerned, although they may not appear in the long term. Second, the method can recognize the key causality patterns which had important regulating effect in the transition process. When other causality patterns converted to these key causality patterns, they tended to convert to themselves rather than other causality patterns. It means that the key causality patterns can control the randomness of the transition process. This feature has important value for investors and decision makers to predict the next state of the international oil market. For example, if the present causality pattern is the key causality patterns, such as P(B(W),D(B,W),M(B,W)) and P(D(B,W),M(B,W)), the next causality pattern would likely be itself. Third, the method can recognize the clustering effect in the transition process. Different clusters showed the inside special structures which had important reference value for predicting the development of the causality among time series. For example, the transition of the causality patterns in cluster 3 was the chain transition but exhibited strong uncertainty in cluster 2. It means if the present causality pattern is in cluster 3, investors and decision makers can refer to the historical path of the fluctuation of short-term causality; if the present causality pattern is in cluster 2, we must prepare several coping strategies (Fig. 9).

To judge the applicability of the proposed method, we have added some simulated experiments and done the additional analysis. By contrast with realistic system, random time series with the lengths as the oil prices were generated following the random walk process. We also generated white noise series with the lengths as the oil prices. To make the time series more general, we respectively generated three groups of four dimensional random time series and three groups of four dimensional white noise series. Both random time series and white noise series represent the simulated system. The analysis of the realistic system and the simulated system were both made based on typological structure indexes of time-varying causality transition network (Table 6). The results showed that there are significant differences between the realistic network and the simulated networks. Realistic network had more nodes and edges. This means the evolution of the relationships between real time series has more diversities, which are caused by the complicated factors in the real world. The modularity class of the realistic network was much higher than the simulated networks. This means the clustering effect is more significant in the evolution of the relationships between real time series. The density, the average clustering coefficient and the average path length showed that nodes connect to each other more tightly in the stochastic networks, which also means the evolution of the relationships between simulated time series has less diversities. These results indicated that the proposed method can reliably uncover the hidden information in the evolution process of the relationship between the real time series, which doesn’t exist in the simulated system.

**Table 6:** The network structure characteristics of the realistic network and the stochastic network.

	Realistic network	Random walk series network 1	Random walk series network 2	Random walk series network 3	White noise series network 1	White noise series network 2	White noise series network 3
The number of nodes	230	99	104	91	96	114	99
The number of edges	1021	329	343	300	262	369	316
Density	0.019	0.034	0.032	0.037	0.032	0.029	0.033
Modularity class	0.293	0.091	0.031	0.03	0.057	0.06	0.122
The average clustering coefficient	0.286	0.346	0.417	0.432	0.321	0.369	0.334
The average path length	4.01	2.88	2.743	2.624	2.872	2.942	3.053

Moreover, we have done the additional analysis to examine the effect of causality thresholds and the length of data. Except the significance level 0.05 in our paper, we chose the significance level 0.01 and 0.1 to determine the causality among time series. Also, we chose different length of data, which respectively covered 3 years (2013–2015), 6 years (2010–2015) and 9 years (2007–2015), to build the multivariate time-varying causality transition networks. All results indicated that the transitions of the causality patterns exhibit similar characteristics. See the details in the Supplementary Information.

The main research purpose of this study is to break the limit of the existing research on univariate time series and bivariate time series and examine the transition behavior of the causality among multivariable time series. The running of a system can be influenced by other systems, however, and the reality is more complex. We will focus on the interaction on different systems in the future research so that we can investigate the inner rules in the entire system. Meanwhile, our method mainly aims to analyze the evolution of the relationships between certain important time series. With the increase of the number of time series, the relationship structures between time series will become more and more complicated. As a result of lacking the same relation patterns at different time, it will be difficult to transfer the evolution of the relation patterns to a network. The results of this paper show that the present method is effective if we study the evolution of the relationships between a few time series. In our future work, we will improve the method and make it more effective when considering more time series into the models.

## Electronic supplementary material

Supplementary Information

## References

[1] Gao XY (2014). Characteristics of the transmission of autoregressive sub-patterns in financial time series. Scientific Reports.

[2] Huang, S. P., An, H. Z., Gao, X. Y., Hao, X. Q. & Huang, X. The Multiscale Conformation Evolution of the Financial Time Series. *Mathematical Problems in Engineering*, 563145, doi:10.1155/2015/563145 (2015).

[3] Gao XY, An HZ, Fang W, Li HJ, Sun XQ (2014). The transmission of fluctuant patterns of the forex burden based on international crude oil prices. Energy.

[4] Huang X, An HZ, Gao XY, Hao XQ, Liu PP (2015). Multiresolution transmission of the correlation modes between bivariate time series based on complex network theory. Physica a-Statistical Mechanics and Its Applications.

[5] Gao ZK, Small M, Kurths J (2016). Complex network analysis of time series. Epl.

[6] Gao ZK, Yang YX, Zhai LS, Jin ND, Chen GR (2016). A Four-Sector Conductance Method for Measuring and Characterizing Low-Velocity Oil-Water Two-Phase Flows. Ieee Transactions on Instrumentation and Measurement.

[7] Wackerbauer R, Witt A, Altmanspacher H, Kurths J, Scheingraber H (1994). A comparative classification of complexity-measures. Chaos Solitons & Fractals.

[8] Szolnoki A, Wang Z, Perc M (2012). Wisdom of groups promotes cooperation in evolutionary social dilemmas. Scientific Reports.

[9] Wang Z, Szolnoki A, Perc M (2013). Optimal interdependence between networks for the evolution of cooperation. Scientific Reports.

[10] Sun XQ, An HZ, Gao XY, Jia XL, Liu XJ (2016). Indirect energy flow between industrial sectors in China: A complex network approach. Energy.

[11] Gao Z-K, Cai Q, Yang Y-X, Dong N, Zhang S-S (2016). Visibility Graph from Adaptive Optimal Kernel Time-Frequency Representation for Classification of Epileptiform EEG. International Journal of Neural Systems.

[12] Li H (2016). Price fluctuation in the energy stock market based on fluctuation and co-fluctuation matrix transmission networks. Energy.

[13] Gao X, Fang W, An F, Wang Y (2017). Detecting method for crude oil price fluctuation mechanism under different periodic time series. Applied Energy.

[14] Zhang J, Small M (2006). Complex network from pseudoperiodic time series: Topology versus dynamics. Physical Review Letters.

[15] Lacasa L, Luque B, Ballesteros F, Luque J, Nuno JC (2008). From time series to complex networks: The visibility graph. Proceedings of the National Academy of Sciences of the United States of America.

[16] Gao ZK, Cai Q, Yang YX, Dang WD, Zhang SS (2016). Multiscale limited penetrable horizontal visibility graph for analyzing nonlinear time series. Scientific Reports.

[17] An HZ, Gao XY, Fang W, Huang X, Ding YH (2014). The role of fluctuating modes of autocorrelation in crude oil prices. Physica a-Statistical Mechanics and Its Applications.

[18] Gao, Z. K., Zhang, X. W., Jin, N. D., Marwan, N. & Kurths, J. Multivariate recurrence network analysis for characterizing horizontal oil-water two-phase flow. *Physical Review E* **88**, doi:10.1103/PhysRevE.88.032910 (2013). 10.1103/PhysRevE.88.032910 24125328

[19] Gao ZK, Yang YX, Zhai LS, Ding MS, Jin ND (2016). Characterizing slug to churn flow transition by using multivariate pseudo Wigner distribution and multivariate multiscale entropy. Chemical Engineering Journal.

[20] Gao ZK (2016). Multivariate multiscale complex network analysis of vertical upward oil-water two-phase flow in a small diameter pipe. Scientific Reports.

[21] Gao ZK, Fang PC, Ding MS, Jin ND (2015). Multivariate weighted complex network analysis for characterizing nonlinear dynamic behavior in two-phase flow. Experimental Thermal and Fluid Science.

[22] Lu X, Su LJ, White H (2017). GRANGER CAUSALITY AND STRUCTURAL CAUSALITY IN CROSS-SECTION AND PANEL DATA. Economet. Theory.

[23] Kahia M, Ben Aissa MS, Lanouar C (2017). Renewable and non-renewable energy use - economic growth nexus: The case of MENA Net Oil Importing Countries. Renewable & Sustainable Energy Reviews.

[24] Konstantakopoulou I, Tsionas MG (2017). The long-run causal relationship between exports and economic growth in the euro area. Applied Economics Letters.

[25] Zanin M, Papo D (2017). Detecting switching and intermittent causalities in time series. Chaos.

[26] Ye H, Deyle ER, Gilarranz LJ, Sugihara G (2015). Distinguishing time-delayed causal interactions using convergent cross mapping. Scientific Reports.

[27] Ghysels E, Hill JB, Motegi K (2016). Testing for Granger causality with mixed frequency data. Journal of Econometrics.

[28] Candelon B, Tokpavi S (2016). A Nonparametric Test for Granger Causality in Distribution With Application to Financial Contagion. Journal of Business & Economic Statistics.

[29] Martinez-Bellver S (2017). Causal relationships between neurons of the nucleus incertus and the hippocampal theta activity in the rat. J. Physiol.-London.

[30] Hong JP (2017). Causal relationship between ICT R&D investment and economic growth in Korea. Technol. Forecast. Soc. Chang..

[31] Lahmiri S (2017). Cointegration and causal linkages in fertilizer markets across different regimes. Physica a-Statistical Mechanics and Its Applications.

[32] Lu FB, Hong YM, Wang SY, Lai KK, Liu J (2014). Time-varying Granger causality tests for applications in global crude oil markets. Energy Economics.

[33] Balcilar M, Gungor H, Hammoudeh S (2015). The time-varying causality between spot and futures crude oil prices: A regime switching approach. International Review of Economics & Finance.

[34] Zeki AKM, Kirca M, Altintas N (2016). The impacts of financial development on growth: A time-varying causality analysis for Turkey. Zb. Rad. Ekon. Fak. Rijeci.

[35] Zeren F, Koc M (2016). Time varying causality between stock market and exchange rate: evidence from Turkey, Japan and England. Ekon. Istraz..

[36] Jammazi R, Ferrer R, Jareno F, Shahzad SJH (2017). Time-varying causality between crude oil and stock markets: What can we learn from a multiscale perspective?. International Review of Economics & Finance.

[37] Granger CWJ (1969). Investigating Causal Relations by Econometric Models and Cross-Spectral Methods. Econometrica.

[38] Gao XY, An HZ, Fang W (2012). Research on fluctuation of bivariate correlation of time series based on complex networks theory. Acta Physica Sinica.

[39] Faes L, Porta A, Nollo G (2015). Information Decomposition in Bivariate Systems: Theory and Application to Cardiorespiratory Dynamics. Entropy.

[40] Faes L (2016). Predictability decomposition detects the impairment of brain-heart dynamical networks during sleep disorders and their recovery with treatment. Philos. Trans. R. Soc. A-Math. Phys. Eng. Sci..

[41] Jia XL, An HZ, Fang W, Sun XQ, Huang X (2015). How do correlations of crude oil prices co-move? A grey correlation-based wavelet perspective. Energy Economics.

[42] Ghoshray A, Trifonova T (2013). Dynamic Adjustment of Crude Oil Price Spreads. Energy Journal.

[43] Blondel, V. D., Guillaume, J. L., Lambiotte, R. & Lefebvre, E. Fast unfolding of communities in large networks. *Journal of Statistical Mechanics-Theory and Experiment*, P10008, doi:10.1088/1742-5468/2008/10/p10008 (2008).

[44] Zhang J, Luo XD, Nakamura T, Sun JF, Small M (2007). Detecting temporal and spatial correlations in pseudoperiodic time series. Physical Review E.

