# Supplementary material for: Reconstructing complex network for characterizing the time-varying causality evolution behavior of multivariate time series
Source: Sci Rep. 2017 Sep 5;7:10486. doi: 10.1038/s41598-017-10759-3 (PMC5585247; doi:10.1038/s41598-017-10759-3)
Supplement: Supplementary file 1 — Supplementary Information [file 41598_2017_10759_MOESM1_ESM.doc]

TITLE:

*Supplementary Information:*

Reconstructing complex network for characterizing the time-varying causality evolution behavior of multivariate time series

AUTHORS AND AFFILIATIONS:

Meihui Jiang^1,2,3^, Xiangyun Gao*^1, 2, 3^, Haizhong An^1,2,3^, Huajiao Li^1,2,3^, Bowen Sun^1,2,3^

1 School of Humanities and Economic Management, China University of Geosciences, Beijing 100083, China

2 Key Laboratory of Carrying Capacity Assessment for Resource and Environment, Ministry of Land and Resources, Beijing 100083, China

3 Key Laboratory of Strategic Studies, Ministry of Land and Resources, Beijing 100812, China

* To whom correspondence should be addressed; E-mail: gxy5669777@126.com

# 1 The examination of the causality thresholds

To examine the effect of causality threshold, we choose three different significance levels as the causality thresholds to determine the causality among time series: 0.01, 0.05 and 0.1. With the increase of the significance level, the number of nodes and edges increases, the densities of networks decrease and the average path lengths increase (Figure S1). As we found at the significance level of 0.05 in our work, the weighted degree of nodes and the weight of edges also follow the power law at the significance levels: 0.01 and 0.1(Figure S2). These results indicate the transition of the causality patterns at different significance levels exhibit similar characteristics.


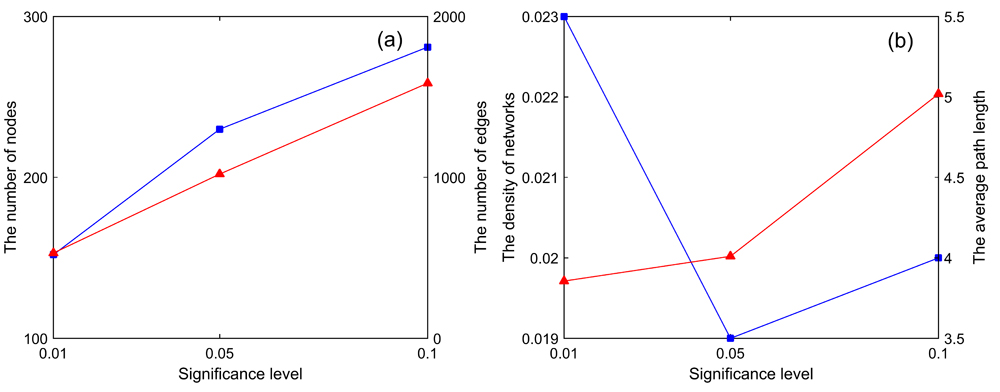


Figure S1. The networks structure characteristics. (a) Number of nodes and edges for different causality thresholds. (b) The density of networks and the average path length for different causality thresholds.


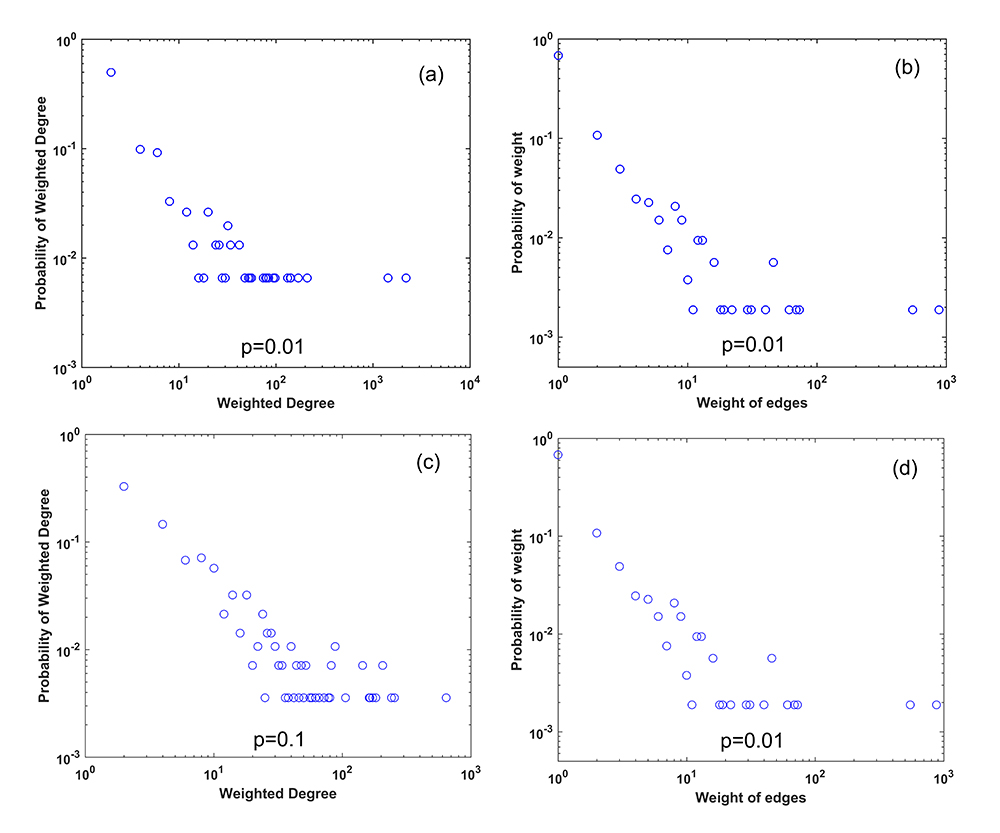


Figure S2. The distribution of weighted degree of nodes and weight of edges of different causality thresholds: 0.01 and 0.1. (a) and (c) are the distribution of weighted degree of nodes. (b) and (d) are the distribution of weight of edges.

# 2 The examination of the length of data

To examine the effect of the length of data, we choose four kinds of crude oil prices, covered the period from 2003 to 2015. The length of data is 13 years. Besides, we choose 3 kinds of length of data to examine our network model: 3 years (2013-2015), 6 years (2010-2015) and 9 years (2007-2015). With the increase of the length of data, the numbers of nodes and edges increase, the densities of network and the average path lengths decrease (Figure S3). The weighted degree of nodes and the weight of edges also follow the power law at the length of data: 3 years, 6 years and 9 year (Figure S4). These results indicate that the length of data would not influence the genuine features of the underlying dynamics in the transition of the causality patterns.


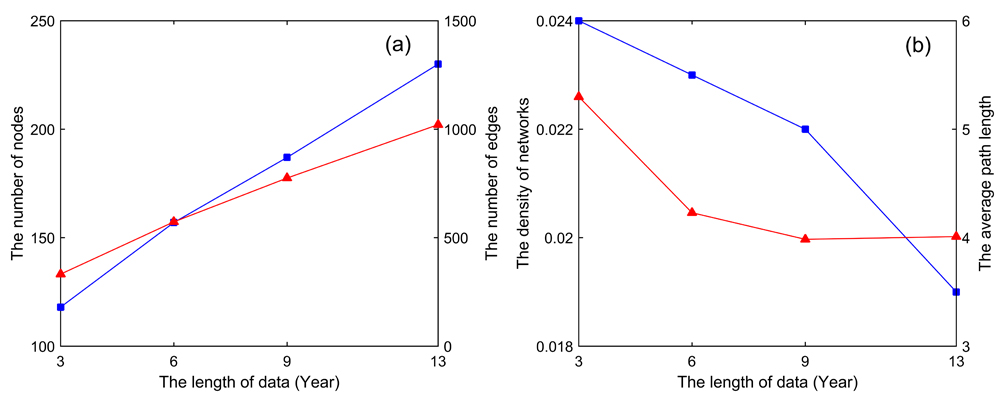


Figure S3. The networks structure characteristics. (a) Number of nodes and edges for different length of data. (b) The density of networks and the average path length for different length of data.


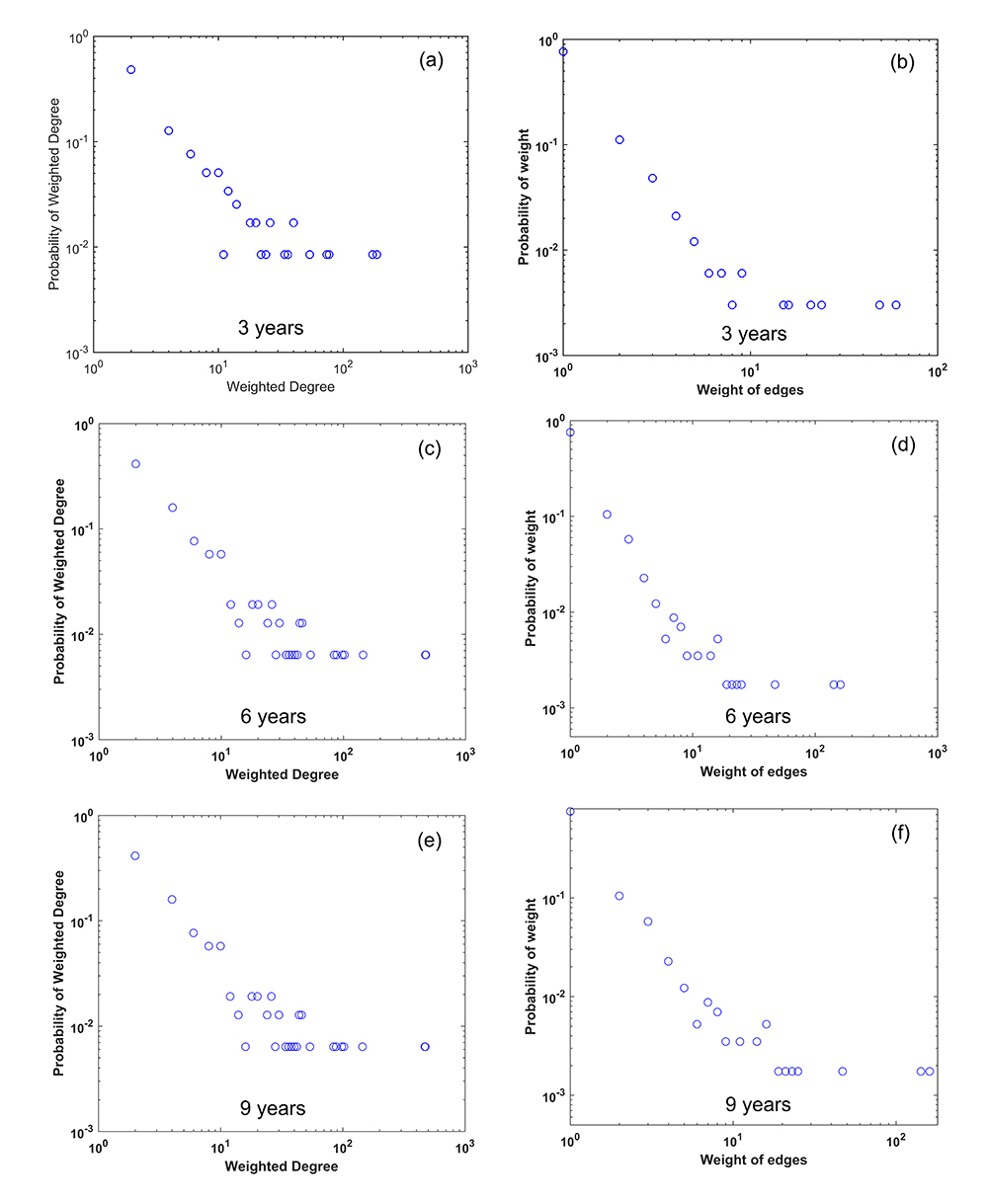


Figure S4. The distribution of weighted degree of nodes and weight of edges of different length of data: 3 years, 6 years and 9 years. (a), (c) and (e) are the distribution of weighted degree of nodes. (b), (d) and (f) are the distribution of weight of edges.

# 3 The examination of the length of sliding window

In this paper, we set the size of a sliding window for 50 days. To examine the effect of the length of sliding windows on finding the dynamics in the transition of the short-term relationship pattern among time series, we choose 3 kinds of length of sliding window to examine our network model: 100 days, 500 days and 1000days. The weighted degree of nodes and the weight of edges follow the power law at the length of sliding window: 100 days, 500 days and 1000days (Figure S5). These results indicate that the length of data would not influence the genuine features of the underlying dynamics in the transition of the causality patterns.


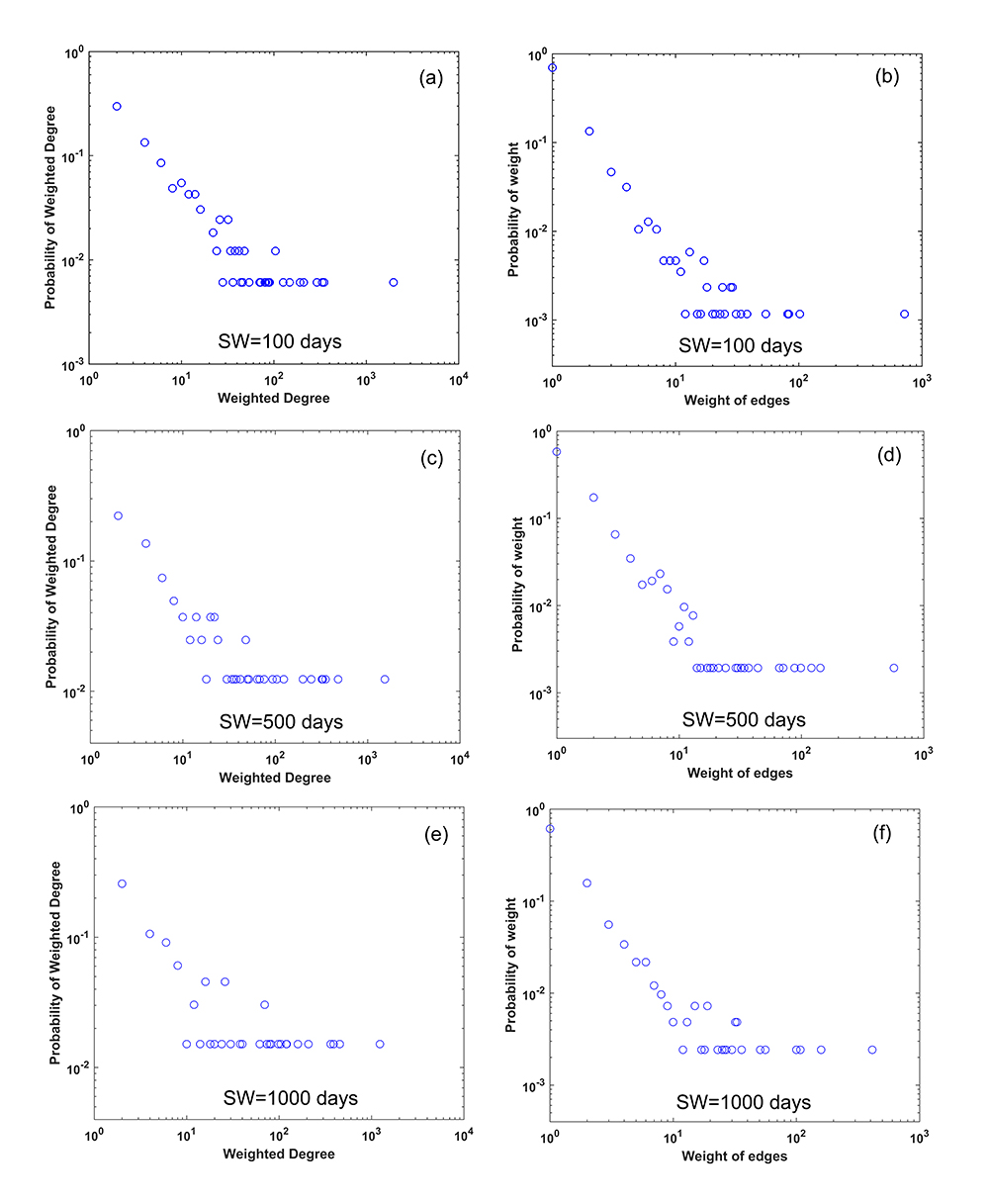


Figure S5. The distribution of weighted degree of nodes and weight of edges of different length of sliding window: 100 days, 500 days and 1000 days. (a), (c) and (e) are the distribution of weighted degree of nodes. (b), (d) and (f) are the distribution of weight of edges.
